# Colorectal cancer patients’ experiences with antineoplastic agents from the perspective of their significant others: a longitudinal qualitative study

**DOI:** 10.1007/s11096-025-01976-2

**Published:** 2025-08-23

**Authors:** Alison Brincat, Antonella Tonna, Patricia Vella Bonanno, Derek Stewart, Anita Elaine Weidmann

**Affiliations:** 1 School of Pharmacy, Applied Sciences and Public Health, Robert Gordon University, Aberdeen, Scotland; 2 Department of Health Systems Management and Leadership, Faculty of Health Sciences, University of Malta, Msida, Malta; 3 School of Pharmacy and Biomolecular Sciences, Royal College of Surgeons, Dublin, Ireland; 4 Institute of Pharmacy, Department of Clinical Pharmacy, Innsbruck University, Innsbruck, Austria

**Keywords:** Antineoplastic agents, Colorectal cancer, Longitudinal study, Patients’ experiences, Significant others

## Abstract

**Background:**

Colorectal cancer and its treatment deeply impact patients' lives. Significant others, often family members, play crucial pre-existing roles in patients’ lives and are closely involved in their care. However, there is limited understanding of the patients’ treatment journey from the significant others’ perspective.

**Aim:**

To explore colorectal cancer patients’ experiences with antineoplastic agents from the perspective of significant others over 24 weeks.

**Method:**

This involved a longitudinal qualitative study with 16 significant others nominated by patients with colorectal cancer. Two in-depth interviews were conducted: one at start and another after 24 weeks of treatment. Interviews were audio-recorded, transcribed and thematically analysed by 2 researchers independently.

**Results:**

Five themes were identified: Patients’ perceptions and knowledge of the illness and treatment, the healthcare system in relation to the illness and treatment, patients’ involvement in treatment decision-making and their experience of medicine-taking, medicine and illness-related impact on patients and others and personal support structures. Significant others observed that patients initially viewed treatment as curative but later perceived as means to extend life-expectancy. While significant others considered that concerns about aesthetics were initially prominent for the patient, fatigue and peripheral neuropathy became most impactful effects. They considered patients' experiences with cancer services to remain overall positive, particularly regarding personalised support from nurse navigators. Significant others noted that early establishment of support network was crucial for fostering resilience throughout the treatment journey.

**Conclusion:**

This study provides insights from significant others highlighting the complex evolution of patients’ experiences and importance of establishing a personalised support network early in treatment journey.

## Impact statements

- The patients’ changing perspective about the intent of treatment with antineoplastic agents shows the need for healthcare professionals to provide clear ongoing communication about treatment goals.
- The value of personalised support to patients by the nurse navigators emphasised the importance of integrating dedicated care coordination roles within cancer care teams to assist patients in navigating complex healthcare systems.
- Healthcare professionals should prioritise proactive management strategies for fatigue and peripheral neuropathy to assist the patients in maintaining better day-to-day functioning.
- Significant others highlighted the need to establish patient’s support network early in the treatment journey to improve patients’ outcomes and coping skills.

## Introduction

Colorectal cancer is among the most common digestive system malignancies, ranking third globally in incidence (10%, 1.9 million cases) and second in mortality (9.4%, 0.9 million deaths) in 2020 [1]. Between 2000 to 2014, European cancer survival rates increased from 54 to 59% for colon cancer and from 51 to 58% for rectal cancer [2]. The journey of patients with colorectal cancer is lengthy and challenging, often involving intensive treatments such as surgery, radiotherapy, antineoplastic agents and targeted therapies. Given the chronic nature of colorectal cancer, patients endure years of treatments that impact their quality of life and place caregiving burdens on family members who face stress, fatigue and work disruptions [3–8].

Understanding the lived experience of patients with cancer undergoing treatment with antineoplastic agents is crucial to provide holistic and person-centred care. Besides the illness itself, treatment with antineoplastic agents often brings physical, emotional and social challenges, including debilitating adverse effects, altered self-perception and disrupted routines. By gaining insights into these experiences, healthcare professionals can better address patients’ needs, optimise symptom management and provide tailored psychosocial support [9, 10].

While previous studies explored the challenges and needs of informal caregivers [11–14], only few studies examined how caregivers perceive and interpret patients’ experiences of cancer and treatment [13, 15]. None of these studies specifically focused on colorectal cancer patients undergoing antineoplastic treatment. This study addresses this gap by exploring the perspectives of significant others (spouse, son/daughter, parent or friend) on the challenges faced by patients along their treatment journey. The term *significant other* is used in this study instead of *caregiver* to reflect the diverse established relationships of those closely involved in patients' lives and care [16–18]. Their insights are crucial to enrich the understanding of patients’ challenges and potentially enhance healthcare services.

### Aim

To explore colorectal cancer patients’ experiences with antineoplastic agents from the perspective of significant others over 24 weeks.

### Ethics approval

This research received approval from the Ethics Review Panel at the School of Pharmacy and Life Sciences, Robert Gordon University (Approval Reference S131) and Health Ethics Committee, at the Ministry for Health, Malta (HEC Reference Number 12/18) in 2018. Governance approval, including data protection, was obtained from hospital administration.

## Method

### Study design

This longitudinal qualitative study used a phenomenological approach, conducting in-depth interviews with significant others at two time-points during the patient’s treatment journey: first interview at start of treatment and another at 24 weeks, upon completing treatment with antineoplastic agents. Phenomenology was considered appropriate since the aim of this research was to explore the lived experiences in-depth, providing insights into the meaning and interpretation of patient’s illness and treatment over time.

### Recruitment of participants

Participants were significant others, nominated by patients undergoing either FOLFOX (parenteral treatment with fluorouracil and oxaliplatin, 12 cycles) or XELOX (oral capecitabine and parenteral oxaliplatin, 8 cycles). FOLFOX regimen is the standard adjuvant treatment for colon cancer, while XELOX is often administered when FOLFOX is less tolerated [19]. Significant others were individuals ≥ 18 years identified by patients as closely involved in their care. Each patient could nominate up to 3 individuals, but all selected only one. Before the interviews, all participants received an information sheet and gave written consent; with verbal confirmation of their continued participation obtained prior second interview. To protect confidentiality, participants were assigned unique codes.

### Development of the interview guide

The interview guide (Table 1) was adapted from the conceptual model by Mohammed et al. [20], which explored the lived experiences of individuals managing an illness and tailored to oncology setting based on insights from a systematic review by Brincat et al. [21]. A pilot study with 4 significant others confirmed feasibility. As no major revisions were required in the interview guide, their data were included in the final analysis. The same interview guide was used at both time-points, with minor adjustments to reflect the timing.

**Table 1:** Interview guide

PLEM major themes from original model [13]	PLEM themes from the adapted model^*^ [14]	Interview questions
Characteristics of significant other		Can you describe what merits you to be nominated as a significant other by the patient?
Medicine—related beliefs	Family members, healthcare professionals, media and culture influence	What does the term ‘chemotherapy’ mean to the patient? Can you describe the thoughts and feelings experienced by the patient when informed that he/she will be starting treatment with chemotherapy? What do you think has shaped his/her thoughts and feelings around chemotherapy?
	General attitude towards medicine	What do you think the benefits of chemotherapy will be?
		What are the patient’s main concerns about starting chemotherapy?
Medicine taking practice	Accepting medicine	Has the patient been explained how he/she shall be receiving the chemotherapy? *How would you describe the patient’s experience of taking chemotherapy?* *Can you tell me how the patient followed the treatment schedule for oral and/or intravenous chemotherapy?*
		Does the patient have a network of support (family members, friends, support groups)? How do they help him/her?
	*Modifying or altering medicine regimen or dose*	*Has the patient ever experienced any dosing adjustment in the treatment by the doctor?*
Medicine- related burden	Medicine characteristics	Are there any aspects of chemotherapy such as the pharmaceutical form (intravenous or oral formulation), colour of size of the tablet, infusion time that might affect the patient?
	Medicine routine	Do you envisage any problems for the patient to follow the treatment schedule as prescribed?
	Medicine adverse events	Do you know whether the patient reported side effects to the doctor or other healthcare professionals?
	Medicine social burden	Do you feel that chemotherapy will have an impact on the patient’s daily life?
	Healthcare associated medicine burden	What are the patient’s experiences with the healthcare system?

^*^Questions in italicised text were included in the interview guide and used for the second interview

### Data generation

Face-to-face interviews with significant others were conducted by the same interviewer (AB) at the National Oncology Centre in Malta between October 2018 and March 2020. Given Malta’s small size, hospital-based oncology services and close-knit nature of the community, ensuring privacy and confidentiality were crucial to encourage participation. Interviews were held in either Maltese or English, according to participant’s preference. The researcher received training in qualitative interviewing to ensure rigour. All interviews were audio-recorded, transcribed verbatim and stored on a password-protected platform. A native Maltese speaker verified a 10% sample of randomly selected transcripts to ensure accuracy in transcription.

### Data analysis

All anonymised transcripts were analysed thematically using framework analysis as outlined by Ritchie and Spencer [22]. The themes and sub-themes identified in a parallel study with patients [23] were applied as a priori codes, with new codes added as necessary. Analysis was performed by the researcher (AB), independently verified by 2 team members (AT, PVB), all native Maltese speakers with qualitative research experience. Any disagreement was resolved by discussion with research team until consensus was reached. Quotations from interviews were translated from Maltese to English (AB) and reverse-translated by another bilingual researcher (AT) to preserve meaning. The study followed the Standards for Reporting Qualitative Research (SRQR) checklist [24] and rigour was ensured through Lincoln and Guba's criteria [25]. Strategies to support trustworthiness included researcher reflexivity and regular team discussions to minimise bias.

## Results

The initial sample size comprised of 12 significant others. In attempt to achieve data saturation, additional participants (n = 4) were recruited bringing the final sample to 16 significant others. The first and second interviews were completed by 16 and 11 significant others respectively. The dropouts occurred due to patient’s discontinuation of antineoplastic treatment (n = 2), patient’s voluntary withdrawal from the study (n = 1) and suspension of interviews due to COVID-19 outbreak (n = 2). Participants were predominantly females (n = 12), related to patient as their spouse (n = 6) with age range between 31 and 72 years (mean 50 ± 14).

Five themes were generated (Table 2); each theme is presented individually and illustrated with relevant verbatim excerpts to help contextualise the data.

**Table 2:** Summary table comparing findings from first and second interview with significant others

Theme	Interview 1 (Start of treatment)	Interview 2 (End of treatment)
Perceptions and knowledge of the illness and treatment	• Patients and significant others lacked experience; perceptions based on personal beliefs • Patients were optimistic about a cure • Knowledge influenced by family and friends	• Views on cancer and treatment evolved with first-hand experience • Patients viewed treatment as life-prolonging. Concerns about recurrence grew • Media (particularly TV) influenced perceptions negatively
The healthcare system in relation to the illness and treatment	• Patients and significant others praised free and coordinated care; some showed distrust due to misdiagnoses and missed follow-up	• Valued dedication of healthcare professionals, particularly oncologists and nurse navigators • Recommended better awareness of services and nurse-led group sessions. Focus shifted to symptom control units and ambulatory chemotherapy
Patient’s involvement in treatment decision-making and their experience of medicine-taking	• Significant others noted patients were passive in treatment decisions, placing full trust in oncologists • Patients were unaware of different treatment options	• Increased awareness about other available treatment options • Realisation that treatment may not meet expectations and may experience adverse effects
Medicine and illness-related impact on patients and others	• Major concerns that treatment will result in hair loss and fatigue resulting in social withdrawal • At diagnosis significant others noted patients to be overwhelmed by fear and intense emotions • Reluctance to disclose diagnosis due to stigma • Almost all patients had to stop working relying primarily on the state for financial support	• Fatigue and neuropathy were the most common experienced adverse effects • As they became familiar with the treatment process, fear gradually gave way to reassurance resulting in improved emotional well-being • Social isolation continued confining their social interactions to the oncology centre where they formed new friendships • The financial burden on patients exceeded what significant others had anticipated, with extra costs to manage adverse effects of treatment
Personal support structure	• Significant others perceived themselves as essential to patients’ support network and felt responsible for their care, with limited support. Patients wanted a single main caregiver • Early coping strategies employed, with health regarded as priority by quitting smoking and increasing physical exercise	• Care by significant others seen as bonding opportunity. Lack of preparation and recognition of significant others by healthcare system • Adaptation focused on day-to- day living and restoring routines post-treatment

### Theme 1: Patients’ perceptions and knowledge of the illness and treatment

At the start of treatment, most significant others lacked prior experience with of colorectal cancer or its treatment, shaping their early perceptions more on personal beliefs than informed knowledge. However, after 24 weeks, their views evolved due to first-hand experiences. Initially, both patients and significant others were anxious when they learned that treatment with antineoplastic agents was necessary post-surgery, previously assuming that surgery alone would suffice. Their concern that cancer may not have been fully resected heightened their emotional and psychological challenges.

“*I was under the impression that it [cancer] had been removed completely [with surgery] so as soon as I learned that she was getting chemo I thought that this was because surgery had not been effective and there is no hope.*” [Participant-14, Interview-1].

Many significant others observed that patients were initially optimistic about the effectiveness of antineoplastic treatment in achieving a cure. However, over time, it became increasingly clear to patients that treatment was more likely to improve life expectancy than offering a definitive cure. Concerns about cancer recurrence intensified towards the end of treatment.

“*She* [the patient] *first agreed to treatment hoping it could cure her, but only recently realising that it is actually stopping it* [cancer] *from growing or spreading further*.” [Participant-8, Interview-2].

“*I know that this improves survival and helps it* [the cancer]* not to grow, by suppressing its growth. However, it seems that she’s [the patient] hoping more for a cure*.” [Participant-5, Interview-2].

Some significant others expressed frustration and were overwhelmed by the volume of information about treatment provided by healthcare professionals during the initial information session. Consequently, significant others prioritised acquiring information that specifically addressed the patients’ weakened immune systems and their increased susceptibility to infections, taking proactive measures to minimise these risks.

“*When we attended the initial information session, I think we got more confused … they were speaking in general … if there are 100 patients with cancer to receive chemo, we’re speaking about 100 different situations*.” [Participant-12, Interview-2].

Patients’ attitudes and knowledge about cancer and its treatment were strongly influenced by past experiences of family and friends. As treatment progressed, they were increasingly influenced by the media (particularly TV), which often had a negative impact on patients. Media may sometimes convey unhelpful messages that induced fear and negatively influenced the public’s view on use of antineoplastic messages.

“*We received my mother’s [cancer] diagnosis soon after my sister finished chemotherapy for breast cancer. Thankfully she* [sister] *was recently informed that she has been cured and is coping quite well. Both of us [the patient and the significant other] are highly optimistic that she [the patient] will have a similar experience*.” [Participant-6, Interview-1].

“*… often the media depicts a patient with cancer as a bald person. I am now aware that this depends on the type of chemo and therefore* [the media] *is creating unnecessary anxiety*.” [Participant-10, Interview-1].

### Theme 2: The healthcare system in relation to the illness and treatment

Significant others generally praised the healthcare system for free services, which eased the financial burden. Most also praised the coordinated and efficient services. Despite this, some negative experiences, such as misdiagnoses and missed follow-up appointments after positive colorectal screening tests, impacted trust in the healthcare system.

“*It was difficult for my wife to regain trust in the healthcare system after she experienced that* incident [patient did not receive the screening test result].” [Participant-9, Interview-1].

Along the treatment journey, the significant others valued the dedication of healthcare professionals, particularly oncologists and nurse navigators, who fostered a holistic care approach.

"*They* [healthcare professionals]* really took care of my husband … They really did everything possible to send him home over Christmas*.” [Participant-7, Interview-1].

Some significant others stressed the need for better awareness of available cancer services, noting that these were often discovered by patients independently. They recommended multiple small nurse-led group sessions along the treatment journey, with information tailored to specific cancer types. Initially, significant others focused their recommendations on the introduction of individualised physical activity programs. Over time, their recommendations focused predominantly on setting up of a symptom control unit for managing complications effectively and provision of ambulatory chemotherapy services.

“*At some point, a symptom control unit should be set up dedicated for oncology patients … it is even cost-effective … When going to the emergency department, we’re occupying beds that can be used for other patients and it is also not safeguarding the immunocompromised patients*.” [Participant-8, Interview-2].

### Theme 3: Patients’ involvement in treatment decision-making and their experience of medicine-taking

Significant others observed that patients played a passive role in decisions about treatment, trusting oncologists without much questioning. This was evident by words such as “*blindly follows”* [Participant-2, Interview-1] and “*accept whatever they offer”* [Participant-16, Interview-1]. However, the significant other of a non-Maltese young patient described that the patient was given the opportunity to choose between treatment options.

“*Personally, I was not involved in the decision…but the doctor* [oncologist] *gave him* [patient] *two options* … *then one week to go home, re-think and then come back to him with a decision on the plan after addressing any queries. He was really helpful and nice*!” [Participant-7, Interview-1].

Some significant others only became aware of other available treatment options at the completion of patient’s treatment. A few patients were noted to be grappling with the realities of their treatment upon realising that treatment may not lead to the desired outcomes or could potentially worsen their condition due to adverse effects. This highlighted the need for more active patient involvement and shared decision-making.

“*I think if there was a choice, all options should be put forward and the patient be allowed to decide*.” [Participant-4, Interview-2].

As treatment progressed, managing adverse effects such as diarrhoea and fatigue at home became difficult. Deciding on when to seek assistance from the hospital emergency department was challenging, often leading to unnecessary visits. At the emergency department, patients waited for a long time and shared rooms with other patients. To avoid this, patients preferred contacting the nurse navigator for advice or reporting symptoms during treatment reviews.

“*Looking back, he experienced diarrhoea quite infrequently. He used to come to A&E after experiencing a single episode of diarrhoea, as he was anxious that it would get worse. The doctors explained that there was nothing to worry about if a single episode … It took us around 2 or 3 visits prior to accepting this advice*.” [Participant-1, Interview-2].

### Theme 4: Medicine and illness-related impact on patients and others

At the start of treatment, significant others noted that patients were primarily concerned about physical adverse effects, particularly fatigue and hair loss, with potential impact on their independence and social life. Despite reassurance from the oncologists that hair loss was unlikely with the specific treatment regimen administered for this indication, this concern persisted among both male and female patients. As treatment progressed, mild physical adverse effects were noted; with peripheral neuropathy and fatigue being the most prevalent complaints.

“*She* [the patient] *was highly concerned about losing her hair when hearing about the treatment. In reality, she only experienced hair thinning … She is experiencing tingling sensation in her hands and feet; but hopeful these resolve now that she has ended her treatment*.” [Participant-5, Interview-2].

At diagnosis, significant others noted patients to be overwhelmed by fear and intense emotions. However, as they became familiar with the treatment process, this fear gradually gave way to reassurance. Many noted that patients initially withdrew from social interactions, most likely due to the stigma associated with cancer.

“*He was lost in thoughts, anxious and uncertain about what was going to happen*.” [Participant-10, Interview-1].

Initially, patients were often hesitant to share their diagnosis with family and friends, sometimes only revealing it when asked about physical changes. Over time, most significant others observed an improvement in patient’s psychological well-being and reduction in fear, although irritability about their future uncertainties lingered.

“*Thankfully he* [the patient] *isn’t keeping everything to himself and isn’t concerned about telling others. The fact that he lost weight often leads to compliments about obtaining a better body shape. Surprisingly he doesn’t seem to be annoyed and usually clearly reveals the truth about his diagnosis*!” [Participant-16, Interview-1].

The social isolation caused by treatment routine, risks of infection and adverse effects led patients to withdraw socially, confining their social interactions to the oncology centre. Patients formed friendships with other patients on the ward, and significant others were concerned that these connections might be lost after treatment.

“*Another thing I’m concerned about is the fact that upon completion of treatment, she will lose the friends she made at the hospital. Her life is going to change, for sure*.” [Participant-14, Interview-2].

Almost all patients had to stop working at the start of treatment, relying primarily on the state for financial support. A significant other appreciated the fact that the employer provided a safe, flexible work environment for the patient. By the end of treatment, the financial burden on patients exceeded what significant others had anticipated, with extra costs for fresh products and medicines to manage adverse effects of antineoplastic agents.

“*Apart from social, psychological and emotional consequences, there were financial ones. We had to buy specific food and personal hygiene products together with medicines, sometimes being bought as prophylaxis*.” [Participant-3, Interview-2].

Most significant others noticed patients turning to religious faith as a coping mechanism, relying on frequent prayers and attending church. Combining medical treatment with religious beliefs gave patients a sense of hope and guidance from a higher power throughout the treatment journey.

*"She* [the patient] *received treatment as recommended, but she was also accompanied by God along the way."* [Participant-12, Interview-2].

### Theme 5: Personal support structure

Significant others perceived themselves as essential to patients’ support networks, often feeling a duty to care for their family members. Whilst most patients wanted a single support provider, significant others felt the need to share the burden with other persons in the patient’s support network. Over time, some significant others viewed caregiving as an opportunity to strengthen their relationship with the patient. The lack of preparation of the significant others for the caregiving role and being overlooked by the healthcare system made the situation challenging.

“*The healthcare system should not overlook the carer…it was difficult [for the significant other] to meet her healthcare team during the ward rounds to discuss these issues*.” [Participant-11, Interview-2].

Coping strategies were employed by patients right from the start. Health became a priority even for the young patient who previously took this for granted by quitting smoking and increasing physical activity. Over time, both patients and significant others tried to adapt to life changes brought about by treatment, focusing on living day by day and hoping to restore their pre-diagnosis routines following completion of treatment.

“*Her wishes are to return to her previous routine, where she does all the chores herself, though she has some limitations and side effects from chemo. But we’ll see as we go through it step by step*.” [Participant-8, Interview-2].

## Discussion

### Statement of key findings

Interviews with significant others revealed that patients often had limited knowledge of colorectal cancer and treatment options throughout their journey. Initially, they viewed antineoplastic treatment as curative, shifting over time to focus on life extension. As they became aware of treatment options, significant others advocated for greater patient involvement in decision-making. Although aesthetic concerns were initially prominent, fatigue and neuropathy later became most impactful. Overall, significant others claimed that patients’ experience with cancer services was largely positive and recommended the introduction of new services such as ambulatory care and symptom management services to enhance holistic care and continuity across treatment.

### Strengths and limitations

This is the first longitudinal qualitative study to capture significant others' perspectives on patients’ experiences with antineoplastic agents for colorectal cancer. Patient-nominated participants offered diverse relationships and viewpoints. An adequate retention rate (69%) was achieved, with 11 participants completing both interviews. One-to-one interviews facilitated open and confidential discussions of personal issues. Measures were taken to ensure research rigour such as developing interview guide based on an adapted conceptual PLEM model, pilot testing, independent data coding by 2 researchers and frequent team discussions.

The study has limitations. Conducted at a single oncology centre in Malta, catering for all cancer patients on the island, findings may not be transferable to other settings. Patient-nominated participants may reflect selection bias, potentially favouring those with more positive views. Despite recruiting additional patients and significant others to achieve data saturation, it was not attained, likely due to heterogeneity among significant others. The small sample size, failure to achieve data saturation and a single-centre setting may limit relevance to other centres. Although data generation occurred between 2018 and 2020, healthcare practices in Malta remained largely unchanged, supporting the relevance of findings. The 24-week interval between interviews could have introduced a degree of recall bias.

### Interpretation of findings

This study is the first longitudinal qualitative exploration of the significant others’ perspective on colorectal cancer patients undergoing antineoplastic treatment. At the start of treatment, both patients and their significant others often had limited knowledge about colorectal cancer and its treatment, leading to misconceptions. These contributed to serious consequences for patients, such as delays in initiation of treatment, unnecessary anxiety and hesitancy. These findings are consistent with a recent systematic review, which identified considerable gaps in patient understanding, including beliefs that colorectal cancer is always fatal and underestimation of risk in asymptomatic individuals [26].

As treatment progressed, patients' understanding shifted from viewing antineoplastic agents as purely curative to recognising its life-prolonging intent. Over time, they became more adept at identifying symptoms and managing adverse effects, highlighting the need for ongoing patient education and support. Significant others emphasised the importance of clear, tailored communications and suggested small-group follow-up sessions to prevent information overload. This is consistent with a cross-sectional study by Mekuria et al. [27] who found that cancer patients prioritised information on patient’s specific cancer type (67%), adverse effects and treatment management (63.3%), followed by prognosis (51.8%).

A noticeable change in patients’ attitudes towards treatment decision occurred over time; shifting from a passive approach at start of treatment to greater desire for shared decision-making as they experienced its impact, with support from their significant others. The lack of patients’ involvement in choice of antineoplastic treatment was also reflected in the study amongst patients [23]. In some countries, such as Malta, a more paternalistic approach prevails with strong familial bonds, respect for medical authority and a hierarchical approach and cancer-related stigma, leading to limited patient involvement in decision-making compared to other Western countries [28–31]. A review by Hubbard et al., which analysed 11 studies across various cancer types found that most patients favoured a collaborative approach throughout the treatment journey [32]. A systematic review of 37 studies found a gap in treatment preferences between patients and physicians, with the former valuing quality of life and the latter focusing on potential adverse effects [33]. Incorporating patient preferences in treatment decisions may enhance satisfaction and builds trust in healthcare professionals [32, 34].

This study showed that patients’ needs evolved throughout treatment, with each individual reacting differently to challenges of the illness based on their personality, coping strategies and understanding of their new situation. Early concerns about their appearance shifted towards managing physical symptoms especially fatigue and chemotherapy-induced peripheral neuropathy (CIPN). CIPN is a common, debilitating adverse effect linked to reduced quality of life and treatment dose reductions [35–40]. Similarly, Pettersson et al. [39] identified numbness/tingling (64%, n = 66) and lack of energy (62%, n = 64) as the most common and distressing symptoms in colorectal cancer based on the Memorial Symptom Assessment Scale. A French multi-centre study reported CIPN in approximately 31%(n = 127) of patients during 5 years after FOLFOX treatment, with the highest prevalence (40%, n = 161) in the first year [40]. Patients in our study were often unaware of such risks, highlighting the need for improved education on anticipating and managing adverse effects. Evidence from other healthcare systems such as acute oncology services in UK shows that dedicated symptom control units can reduce symptom burden and prevent unnecessary emergency visits [41–43]. Despite this, further research is warranted to assess their feasibility within the Maltese healthcare system.

Cancer-related stigma also impacted patient’s social well-being, with many being hesitant to disclose their diagnoses to others. While international studies highlight post-treatment distress linked to fear of recurrence, existential concerns and employment changes [44–47], our findings suggest that stigma begins early in the treatment journey. Addressing these issues require timely psychosocial interventions and public awareness campaigns. Attention to mental health should begin at the point of diagnosis, with personalised psychological support playing a vital role [48, 49].

Significant others noticed that patients adjusted their routines around treatment cycles over time, which enhanced resilience and improved coping strategies. Spirituality and faith were crucial coping mechanisms in helping patients manage their emotions from the early stages of treatment. A study by Khan et al. [50] found that older cancer patients were more likely to find solace in religion compared to younger patients, using it to manage fear and accept end-of-life considerations. Similar to our study, a systematic review by Okere et al. [51] analysing 35 studies, found that social support interventions (48.6%, n = 17) were most used in comparison to spiritual interventions (5.7%, n = 2) to improve the quality of life for individuals with colorectal cancer. In our study, significant others highlighted the necessity to establish patient support network early in the treatment journey to improve patients’ outcomes and coping ability. Healthcare professionals should be aware of patients’ spiritual and social needs and how these may evolve during treatment to enhance personalised care and improve patient well-being [52, 53].

Financial concerns became increasingly significant as treatment progressed. Significant others noted that working-age patients felt the impact of reduced income, echoing research that younger cancer survivors face greater financial hardship than those over 65 (28.4% vs. 13.8%) due to lower savings and higher financial responsibilities [54]. Addressing this issue early by providing referrals to financial counselling and support services could help alleviate the burden.

### Further research

Further research is needed to evaluate effectiveness of educational interventions in enhancing patients' and significant others' understanding of treatment with antineoplastic agents and to assess support programs tailored to significant others. Additionally, studies should explore impact of cancer-related social stigma on patients' quality of life and treatment outcomes as well as patients’ and healthcare professionals' perspectives on the role of significant others in cancer care.

## Conclusion

Significant others’ perspectives reveal patients’ evolving needs and highlight the importance of involvement of significant others from the time of initiation of treatment. Despite their crucial role, significant others often go unrecognised by the healthcare system. Healthcare professionals should promote open communication about illness and treatment options and provide educational resources or training that better equip significant others to support patients throughout treatment, contributing to more holistic cancer care.
